# Imaging of Caseous Mitral Annulus Calcification

**DOI:** 10.1016/j.jaccas.2024.102979

**Published:** 2024-12-18

**Authors:** Roni Levin, Orly Goitein, Elena Bekker, Yafim Brodov, Haim Wexler Dov, Avigdor Bar Sef, Adi Butanero, Leonid Sternik, Rafael Kuperstein, Sagit Ben Zekry

**Affiliations:** a Department of Medicine “B,” Sheba Medical Center, Ramat Gan, Israel; b Tel Aviv University, Tel Aviv, Israel; c Diagnostic Imaging, Sheba Medical Center, Ramat Gan, Israel; d Cardiovascular Imaging, Leviev Heart Center, Sheba Medical Center, Ramat Gan, Israel; e Heart Failure Unit, Kupat Holim Clalit, Dan Petach Tikva, Israel; f Cardiology Department, Meir Medical Center, Ramat Gan, Israel; g Non Invasive Cardiology Unit, Leviev Heart Center, Sheba Medical Center, Ramat Gan, Israel; h Cardiothorasic Department, Leviev Heart Center, Sheba Medical Center, Ramat Gan, Israel

**Keywords:** caseous mitral annulus calcification, cardiac computed tomography angiography, echocardiography

## Abstract

We present 2 cases of caseous mitral annulus calcification (MAC) in which one patient was asymptomatic whereas the second experienced left hemianopsia. Both patients underwent transthoracic and transesophageal echocardiography exams which revealed severe MAC with a mass consistent with caseous MAC. A mobile component of the caseous MAC was observed in the patient with left hemianopsia. Cardiac computerized tomography angiography confirmed the diagnosis. Considering the high surgical risk of caseous MAC debridement, both patients were treated conservatively with uneventful clinical follow-up.

## Graphical Abstract

**Figure:**
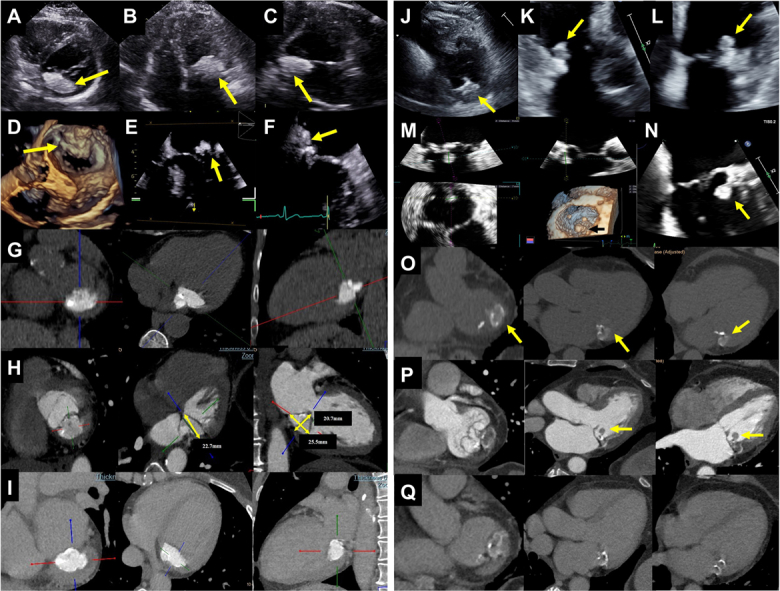

## Case 1

**Figure 1:**
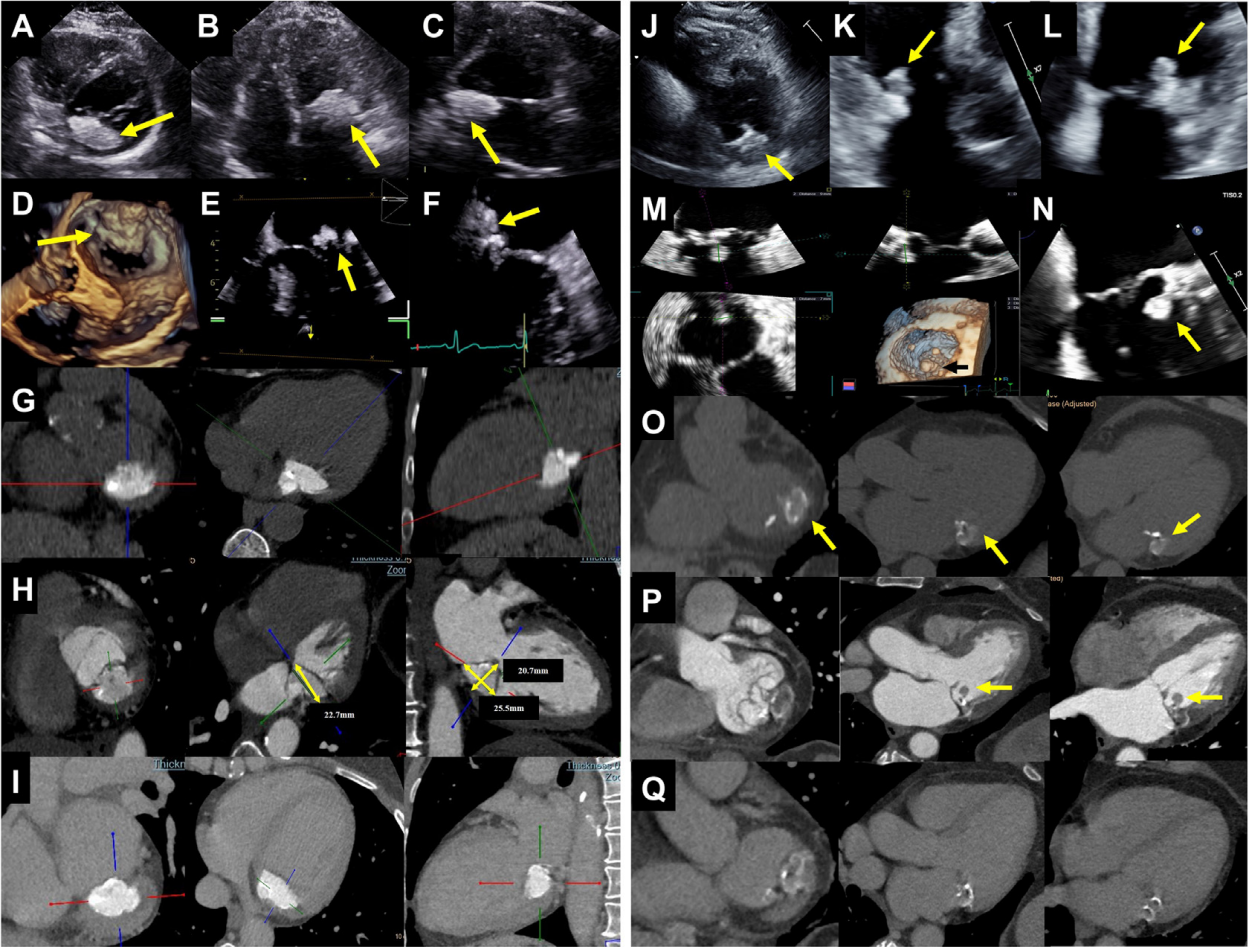
Multimodality Images Case 1 is presented in A to I in which short-axis, 4-chamber, and 2-chamber views are shown, respectively, with (A to C) transthoracic echocardiography (TTE), (D-F) transesophageal echocardiography (TEE), (G) multiplanar reconstrication (MPR) noncontrast cardiac computed tomography angiography (CTA), and (H) arterial and (I) venous phases. TTE short-axis view at the mitral valve annulus (MA) level shows an inferior-posterior calcified mass (A, yellow arrow). The mass is extended towards the left atrium as shown in the 4-chamber view (B, yellow arrow) and 2-chamber view (C, yellow arrow). The surgical view on 3-dimensional TEE (D, yellow arrow) and 2-dimensional 4-chamber (E, yellow arrow) and 2-chamber view (F, yellow arrow) emphasize the irregularity of the mass while protruding to the left atrium. Multiplanar cardiac CTA images with noncontrast, arterial and venous phase, are presented in G, H, and I, respectively. In noncontrast images, the caseous mitral valve annulus calcification (MAC) is hyperattenuated with nonhomogenous calcification, especially seen in the edges of the mass. Note the relatively hypodense core. The caseous MAC protrude extends both towards left atrial and ventricle and was measured in arterial phase 22.7 × 20.7 × 25.5 mm (H). Enhancement is not seen in both arterial and venous phase (H, I). Case 2 is presented in J to Q in which short-axis, 3-chamber, and 4-chamber views are shown, respectively, with TTE (J to L), TEE (M and N), noncontrast MPR cardiac CTA (O), and arterial (P) and venous phases (Q). The TTE short-axis view of the left ventricle shows a caseous MAC extension toward the left ventricle (J, yellow arrow). The attached mobile portion is shown in K and L (yellow arrow) at 3-chamber and 4-chamber views, respectively. The TEE multiplanar 3-dimensional TEE measuring the mobile component of the caseous MAC (9 × 7 × 10 mm) and a ventricle view of it is shown in M (black arrow). A 2-dimensional TEE, 4-chamber view of the caseous MAC including the mobile mass is shown in N. Establishment of caseous MAC diagnosis with cardiac CTA is shown in O to Q. (O) Shows the nonhomogenous calcified MA mass (yellow arrow) with noncontrast images. Note the relatively hypodense core. The mobile mass is better defined with the contrast images (P, yellow arrow). No mass enhancement is observed in both arteria (P) and venous (Q) phases.

A 61-year-old woman was referred for stress echocardiography due to atypical chest pain. The transthoracic echocardiograph (TTE) revealed severe mitral annulus calcification (MAC) with a calcified mass measuring 33 × 33 mm attached to the posterior aspect of the mitral valve annulus (MA) which was further extended towards the left ventricle and atrium (Figures 1A to 1C, yellow arrows; Video 1) consistent with a caseous MAC. Imaging with transesophageal echocardiography (TEE) emphasized the mass irregularity and the extension towards left atrium and ventricle (Figures 1D to 1F, yellow arrows). Diagnosis was confirmed with cardiac computed tomography angiography (CTA). Noncontrast images showed a hyperdense mass on the posterior aspect of the MA with hypodense core and peripheral calcifications (measured up to 790 HU) (Figure 1G). The mass sized 22.7 × 20.7 × 25 mm in arterial phase (Figure 1H, Video 2). No evidence of contrast enhancement was noted in both arterial (Figure 1H) and venous phase (Figure 1I).

**Take-Home Messages**

• Multimodality imaging is essential for caseous mitral annulus calcification diagnosis.
• Echocardiography should be the first modality whereas cardiac computed tomography angiography is the most important complementary tool.

## Case 2

A 69-year-old woman was admitted with persisting left hemianopsia which was diagnosed as retinal artery occlusion. The TTE revealed severe MAC in the posterior aspect of the MA, mimicking intracardiac tumor which is consistent with caseous MAC diagnosis. The mass extended towards the left ventricle with a mobile component measured 9 × 7 × 10 mm (Figures 1J to 1L, yellow arrows; Video 3). This was better defined with 3-dimensional TEE (Figure 1M, Video 4) and 2-dimensional TEE (Figure 1N, yellow arrow), The diagnosis was established with cardiac CTA; in noncontrast imaging, a nonhomogenous calcified mass was observed in the posterior aspect of the MA extending towards the left ventricle (Figure 1O, yellow arrows). The mass size was 27 × 15 × 22 mm, its core measured 48 HU, and its periphery measured 790 HU. Following contrast administration, no contrast enhancement was noted (Figure 1P, Video 5). In the ventricular aspect, a well-defined mobile mass was attached to the annular mass (Figure 1P, yellow arrows; Video 5). Late phase imaging (venous) revealed no enhancement (Figure 1Q). It was concluded that the retinal artery occlusion was most probably an embolus from the caseous MAC.

MAC is a degenerative disorder affecting the cardiac fibrous annulus of the mitral valve.^1^ Caseous (also called “toothpaste”) MAC is a rare form of MAC characterized by central liquefaction necrosis consisting of amorphic eosinophilic acellular fluid; the surrounding rim contains macrophages and lymphocytes as well as multiple areas of calcification, fatty acid, cholesterol, and necrotic zone.^2^ The diagnosis should be made using a multimodal imaging approach where echocardiography is usually the first imaging tool.^3^ It is typically found in the posterior MA or periannular region with an echodense peripheral appearance and a central area with echolucency. It can extend towards the left atrium or the left ventricle.^1^^,^^3^ Cardiac CTA is the most important complementary imaging tool. In noncontrast imaging, a hyperdense mass with peripheral calcification (measured >600 HU) and a hypodense core (measured <50 HU) is observed. Following contrast administration, no enhancement is expected in both early (arterial) and late imaging (venous) phase.^3^ Considering the high surgical risk associated with caseous MAC, both patients were treated conservatively with uneventful clinical follow-up.

## Funding Support and Author Disclosures

The authors have reported that they have no relationships relevant to the contents of this paper to disclose.

## Appendix

Video 1 TTE short-axis view at the mitral valve level, not the caseous MAC.

Video 2 CCTA, multiplanar imaging, full cardiac cycle in arterial phase demonstrating the caseous MAC.

Video 3 TTE Chamber view of the caseous MAC.

Video 4 3D TEE surgical view (left) and ventricle (right) view of the caseous MAC.

Video 5 CCTA, multiplanar imaging, full cardiac cycle in arterial phase demonstrating the caseous MAC and its mobile component.
